# Initial Surgical Management of Penetrating Neck Trauma With an Esophageal Injury: A Case Report

**DOI:** 10.7759/cureus.88705

**Published:** 2025-07-24

**Authors:** Alfredo S Abarca Magallon, Luis Ivan Parada Longoria, Andrea Rodriguez Cisneros, Jorge Avila Vazquez, Jorge Gonzalez Arteaga

**Affiliations:** 1 Department of Colorectal Surgery, Hospital Regional Adolfo López Mateos, Instituto de Seguridad y Servicios Sociales de los Trabajadores del Estado (ISSSTE), Mexico City, MEX; 2 Department of Surgical Oncology, Hospital General de León, León, MEX; 3 Department of General Surgery, Hospital General de León, León, MEX; 4 Department of General Surgery, Hospital General de Penjamo, Penjamo, MEX

**Keywords:** esophageal injury, penetrating trauma neck, primary surgical repair, surgical case reports, trauma surgery outcomes

## Abstract

Esophageal perforation secondary to penetrating trauma is rare but potentially life-threatening and associated with high morbidity. We present the case of a 24-year-old male who arrived at the emergency department with a penetrating neck wound from a firearm projectile. He was hemodynamically stable upon arrival, and imaging studies suggested esophageal injury. Surgical exploration confirmed a Grade III esophageal laceration involving more than 50% of the anterior wall. Primary repair with a two-layer closure and an omohyoid muscle flap was performed. The postoperative course was favorable, with no evidence of fistula or leakage on esophagogram. The patient resumed oral intake and was discharged in good condition seven days post-trauma. This case highlights the importance of early recognition, imaging, and aggressive surgical management of esophageal injuries in penetrating neck trauma to reduce morbidity and improve outcomes.

## Introduction

Esophageal perforations secondary to trauma are rare; however, they represent one of the most complex surgical emergencies due to their high morbidity and mortality, which can reach up to 30% if diagnosis and treatment are delayed [1]. The most common mechanism of injury is gunshot wounds (70-80%), followed by sharp object injuries (15-20%) [2]. The cervical esophagus is the most frequently affected segment. Typical clinical signs include local pain, dysphagia, subcutaneous emphysema, neck stiffness, dysphonia, salivary leakage through the wound, hematemesis, and hemoptysis. Esophageal trauma is often accompanied by other injuries, such as those to the airway, vascular structures, spinal cord, or thorax [3].

The evaluation of penetrating neck trauma should follow the ABCDE protocol of Advanced Trauma Life Support (ATLS), prioritizing airway protection and cervical spine stabilization, and taking into account the mechanism of trauma, associated injuries, and the clinical condition of the patient to guide further management [4].

Surgical management should be aggressive and guided by key principles: identification of the injury, removal of devitalized tissue, primary closure of the defect, preferably with muscle flaps, and adequate drainage to prevent complications [5].

## Case presentation

A 24-year-old man with a history of marijuana, cocaine, and methamphetamine use for eight years presented to the emergency department with a penetrating neck wound caused by a firearm projectile. Upon arrival, the patient was managed according to ATLS protocol. He had a patent airway, no stridor, and anterior cervical subcutaneous emphysema. Breath sounds were diminished on the right side, with no crepitus on palpation. His oxygen saturation was 92%. He was hemodynamically stable with a blood pressure of 120/70 mmHg, a mean arterial pressure of 86 mmHg, and a heart rate of 120 bpm. No active bleeding or expanding hematomas were noted. Neurologic exam showed a Glasgow Coma Score of 15 with equal and reactive pupils.

**Figure 1:**
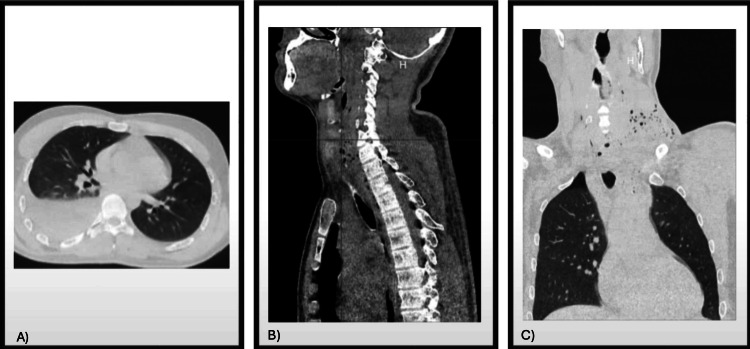
(A) Axial section of a simple and contrast-enhanced chest CT scan showing the presence of a right-sided hemopneumothorax, with evident air-fluid level and associated pulmonary contusion. (B) Sagittal view of a simple and contrast-enhanced CT scan of the neck and thorax. (C) Coronal view of a simple and contrast-enhanced CT scan of the neck and thorax. In both sections (B and C), subcutaneous emphysema is observed along the bullet trajectory through the cervical region, without evidence of vascular or spinal lesions. Additionally, image 1C also shows the right-sided hemopneumothorax.

Physical examination revealed an entry wound in the anterior triangle of the left neck (Zone II) and an exit wound in the posterior right thoracic region, without evidence of injury elsewhere. Due to his stable condition, a contrast-enhanced CT scan of the neck and thorax was obtained (Figure 1A, 1C), revealing a right-sided hemopneumothorax with 510 cc of blood evacuated upon chest tube placement. No ultrasound was performed; diagnosis was based on chest CT, which revealed right-sided hemopneumothorax. Additional findings included a right apical pulmonary contusion, subcutaneous emphysema, and strong suspicion of esophageal injury without vascular or spinal involvement.

**Figure 2:**
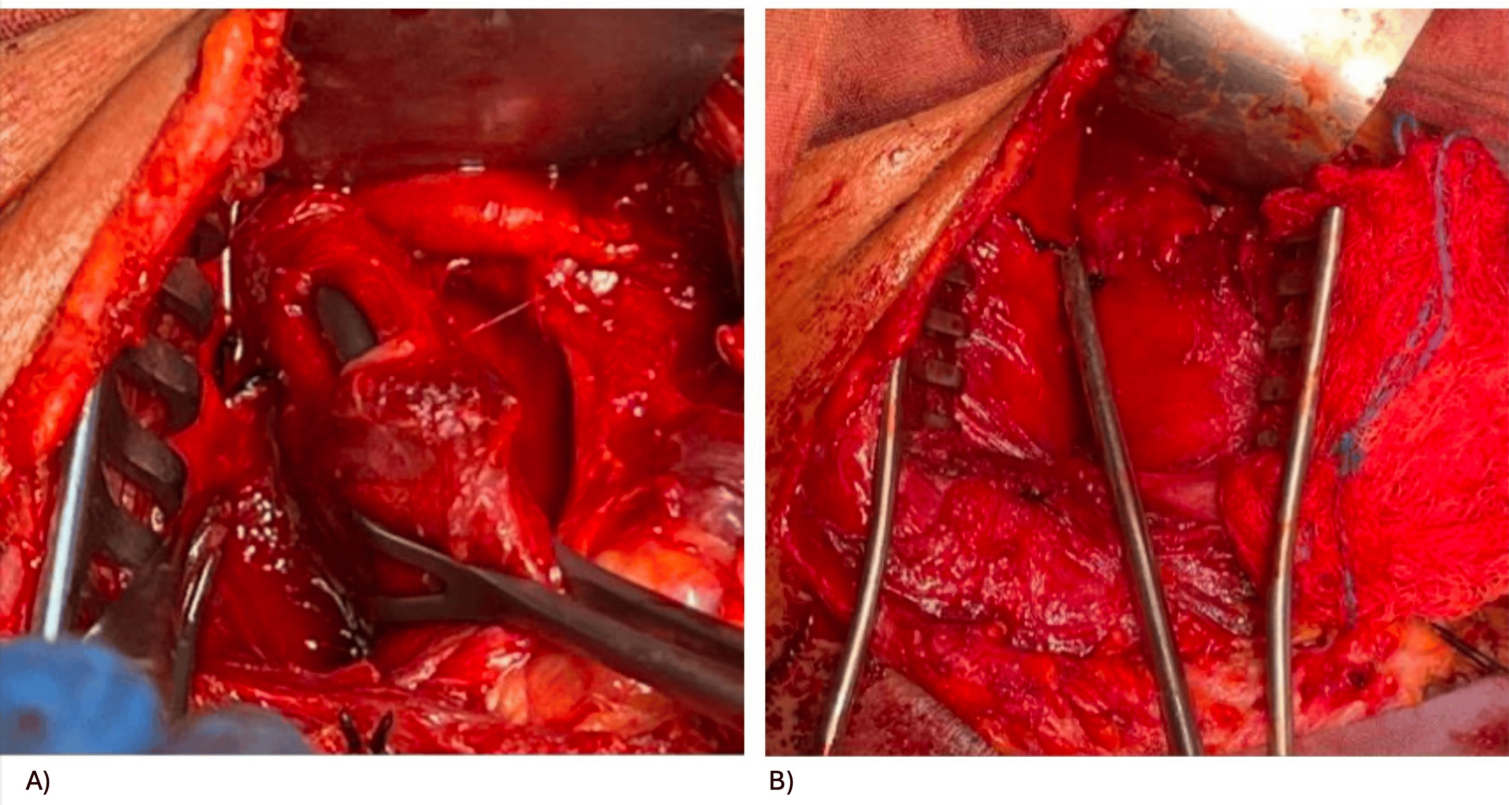
(A) Evidence of AAST grade III lesion on the anterior aspect of the esophagus. (B) Primary raffia of the esophagus in two planes with muscle flap patch. AAST: American Association for the Surgery of Trauma

The patient was taken to the operating room for cervical exploration. A transverse cervical incision revealed a grade III anterior esophageal injury (over 50% of the circumference), according to the American Association for the Surgery of Trauma classification (Figure 2). A primary two-layer repair was performed: inner mucosal layer with 3-0 Monocryl and an outer muscular layer with an omohyoid muscle flap. A closed drain was placed, and the wound was closed in layers.

**Figure 3:**
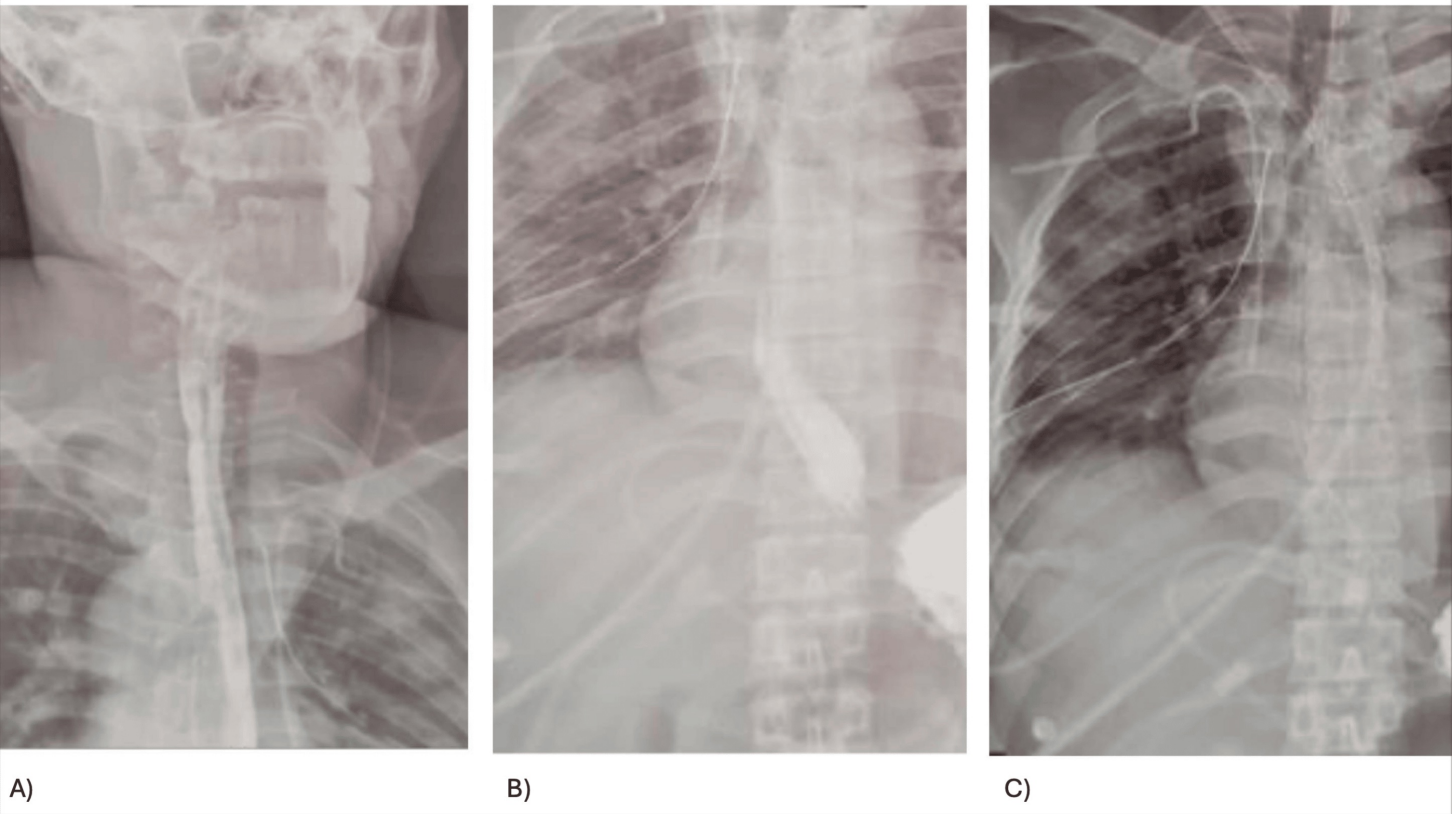
(A, B) Esophagogram with no evidence of alterations in swallowing mechanics, no pharyngonal reflux, esophagus with adequate passage of contrast medium, no sites of stenosis or contrast leakage. (C) Stomach showing normal morphology, mucosa without thickening of the wall, or ulcerative images.

Postoperatively, the patient was managed with broad-spectrum antibiotics, intravenous analgesia, and enteral fasting. He was extubated on postoperative day 1 and discharged from the ICU on day 2. On day 7, the endopleural tube was removed, and an esophagogram showed no contrast leakage or fistula (Figure 3). Oral feeding was restarted and tolerated. He was discharged home in good condition with no complications.

## Discussion

Penetrating neck injuries account for 5-10% of all traumatic injuries in adults and represent a rare cause of esophageal perforation [1]. While iatrogenic causes account for the majority of esophageal injuries (59%), only 4% are due to trauma [2]. These injuries are associated with a high mortality rate of up to 20%, which increases significantly if diagnosis and treatment are delayed. Morbidity is also high due to frequent involvement of adjacent structures, such as the trachea (up to 75% of cases), and potentially vascular, spinal, or thoracic injuries [3].

The cervical esophagus is most frequently affected, especially in Zone II, where vital structures such as the esophagus, trachea, jugular veins, and carotid arteries are located. Zone I injuries, though less frequent, are associated with higher mortality due to their proximity to the mediastinum [4].

Management of these patients should follow ATLS principles, considering the mechanism of injury and hemodynamic status [5]. In stable patients, imaging studies such as cervical and thoracic CT scans are recommended to assess potential injuries, although CT has limited sensitivity for esophageal trauma [6]. Additional studies, such as contrast esophagography or endoscopy, may be used to confirm esophageal injury, with reported sensitivities up to 100% and 92.4%, respectively [7].

In unstable patients or those with hard signs of aerodigestive or vascular injury, surgical exploration should not be delayed, as treatment initiated more than 24 hours after injury significantly increases mortality [8].

Most esophageal injuries are classified as grades I-III, which are amenable to primary repair [9]. Grades IV and V typically require staged procedures such as diversion and delayed reconstruction [10].

Surgical principles include early and aggressive intervention, complete debridement of necrotic tissue, primary repair reinforced with vascularized flaps, and adequate drainage [11]. Postoperative care involves enteral fasting, broad-spectrum antibiotics, and close monitoring for complications such as pneumonia, anastomotic leakage, or recurrent laryngeal nerve injury. Among these, anastomotic leaks are the most severe, with mortality ranging from 2-12% [12].

This case reinforces current evidence supporting prompt recognition and intervention in esophageal injuries. A structured diagnostic and surgical approach is essential to achieving favorable outcomes in patients with penetrating neck trauma.

## Conclusions

Although rare, traumatic esophageal injuries are life-threatening and associated with high morbidity due to the potential involvement of multiple cervical and thoracic structures. Early recognition based on clinical suspicion and imaging, along with prompt and aggressive surgical intervention, is essential to improving patient outcomes. While broader recommendations cannot be drawn from a single case, our experience supports the potential value of having institutional protocols in place to guide the evaluation and management of penetrating neck trauma and reduce diagnostic delays and complications.
